# Nano-Enhanced Cancer Immunotherapy: Immunology Encounters Nanotechnology

**DOI:** 10.3390/cells9092102

**Published:** 2020-09-15

**Authors:** Ernesto Bockamp, Sebastian Rosigkeit, Dominik Siegl, Detlef Schuppan

**Affiliations:** 1 Institute of Translational Immunology, University Medical Center, Johannes Gutenberg University Mainz, 55131 Mainz, Germany; bockamp@uni-mainz.de (E.B.); srosigke@uni-mainz.de (S.R.); dosiegl@students.uni-mainz.de (D.S.); 2 Research Center for Immunotherapy, University Medical Center, Johannes Gutenberg University Mainz, 55131 Mainz, Germany; 3 Division of Gastroenterology, Beth Israel Deaconess Medical Center, Harvard Medical School, Boston, MA 02215, USA

**Keywords:** immune checkpoint inhibitor, CAR T cell therapy, bi-specific antibody therapy, tumor microenvironment, macrophage, myeloid derived suppressor cells (MDSC), PD-1, PD-L1, siRNA, toll like receptor (TLR)

## Abstract

Cancer immunotherapy utilizes the immune system to fight cancer and has already moved from the laboratory to clinical application. However, and despite excellent therapeutic outcomes in some hematological and solid cancers, the regular clinical use of cancer immunotherapies reveals major limitations. These include the lack of effective immune therapy options for some cancer types, unresponsiveness to treatment by many patients, evolving therapy resistance, the inaccessible and immunosuppressive nature of the tumor microenvironment (TME), and the risk of potentially life-threatening immune toxicities. Given the potential of nanotechnology to deliver, enhance, and fine-tune cancer immunotherapeutic agents, the combination of cancer immunotherapy with nanotechnology can overcome some of these limitations. In this review, we summarize innovative reports and novel strategies that successfully combine nanotechnology and cancer immunotherapy. We also provide insight into how nanoparticular combination therapies can be used to improve therapy responsiveness, to reduce unwanted toxicity, and to overcome adverse effects of the TME.

## 1. Introduction

Cancer immunotherapy can provide powerful and long-lasting anti-cancer responses in patients with advanced or metastasized tumors that are otherwise resistant to conventional therapy [1]. Mechanistically and illustrated by the clinical efficacy of immune checkpoint inhibitors (ICIs), cancer immune therapies aim to increase the overall fitness of the immune system by interfering with key immune regulatory mechanisms [2]. As exemplified by chimeric antigen receptor (CAR) T cell therapies, a second powerful mode of action for immunotherapies is to redirect the destructive power of adaptive immune cells towards patient-specific tumor targets [3]. Despite the undisputed clinical efficacy and long-term response rates of immunotherapies observed in various cancer types, the majority of patients receiving treatment will not benefit from immunotherapy and some initially responding patients will eventually relapse [4,5]. In addition and owing to the enhanced immune responses and potential severe off-target effects, significant immune toxicities have been observed in patients receiving therapies with ICIs and CAR T cells [6].

**Figure 1:**
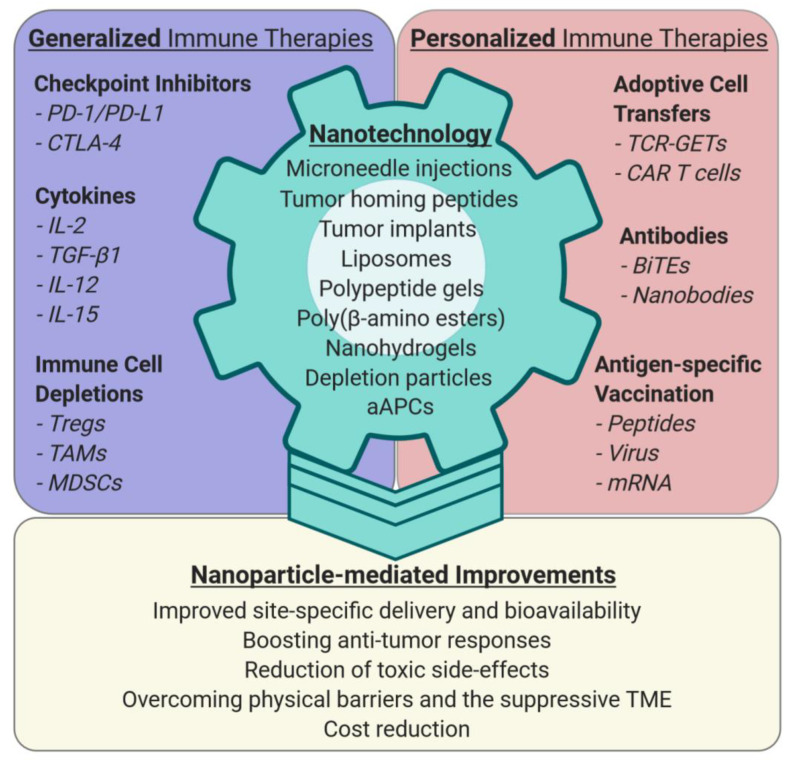
Nanotechnology to improve general and personalized cancer immunotherapies. Nanoparticles can guide given therapeutic agents to specific sites in the body via systemic application, tumor implants, microneedle injection, or tumor homing peptides to improve their bioavailability and stability. Nanomaterials with in vivo efficacy and tolerability are, for example, liposomes, polypeptide gels, poly-β-amino esters, nanohydrogels, or guided aAPCs (artificial antigen presenting cells). They can be engineered to deplete or inhibit immune cell subtypes. Nanoparticle-enhanced efficacy of immune therapies can result in better anti-tumor responses, reduction of systemic toxicities, and cost reduction, because lower amounts of expensive immunotherapeutic agents are needed to achieve a comparable or superior therapeutic effect. Moreover, nanoparticle-mediated targeting of immune suppressive cell types in the TME (tumor microenvironment), especially myeloid cells (TAMs, MDSCs), can make solid tumors more accessible to T- and cancer cell-directed immunotherapy. Abbreviations: programmed cell death protein 1 (PD-1), programmed cell death 1 ligand 1 (PD-L1), cytotoxic T-lymphocyte-associated protein 4 (CTLA-4), interleukins (IL), regulatory T cell (Treg), tumor associated macrophage (TAM), myeloid-derived suppressor cell (MDSC), T cell receptor genetically engineered T cells (TCR-GETs), chimeric antigen receptor (CAR), artificial antigen presenting cell (aAPC).

Extensive preclinical research and first clinical data demonstrate that nanotechnology can overcome some of the challenges that currently limit cancer immunotherapy (Figure 1).

However, broadening the clinical applicability of cancer immunotherapy with the help of nanotechnology requires an improved understanding of the mechanisms limiting cancer immune treatment [4,7]. For example, nanotechnology cannot overcome tumor-intrinsic resistance factors such as the complete lack of T cell recognition owing to missing or lost tumor antigens. Nonetheless, other resistance mechanisms like the absence of factors needed for immune cell attraction and stimulation; the inability to deliver, release, and stimulate immune cells to an inaccessible and immunosuppressive tumor microenvironment (TME); and the danger of developing severe immune toxicities can be completely overcome or mitigated by nanotechnology.

## 2. Nano-Enhancing Generalized Immune-Boosting Cancer Therapies

Generalized immune-boosting therapies improve the overall fitness of immune cells and aim to initiate killing of cancer cells, previously spared by the immune system. In contrast to personalized cancer therapies that target patient-specific oncogenic vulnerabilities, generalized immune-boosting therapies do not require prior knowledge of individual tumor-specific gene mutations or patient-specific immune characteristics such as human leukocyte antigen (HLA) polymorphisms. The first generalized immune-boosting anti-cancer therapy dates back to the year 1891, when William B. Coley started to treat bone and soft-tissue cancer patients with bacterial toxins [8]. Despite the success of Coley’s bacterial toxins in some cancer patients, general molecularly defined immune-boosting therapies only recently found their way into standard clinical application.

### 2.1. Cytokines

Initial modern-day immune-boosting therapies used systemic application of interferon-α (IFN-α) or interleukin-2 (IL-2) and proved that injection of pro-inflammatory cytokines could induce durable anti-tumor immune responses in some cancer types [9,10]. This initial accomplishment was followed by the demonstration that injection or modulation of additional cytokines such as IL-10, -12, -15, and -21; granulocyte-macrophage colony-stimulating factor (GM-CSF); tumor necrosis factor (TNF); and transforming growth factor-β1 (TGF-β1) could equally promote favorable therapy responses in some patients [11,12]. Although cytokine therapies clearly enhanced anti-cancer immune responses, clinical efficacy was generally low, limited to certain cancer types, and came at the cost of unwanted toxic side effects. To increase clinical utility and to make cytokine-based treatment safer, several nanoparticle-based delivery systems were developed. Noteworthy, preclinical approaches are, for example, packaging of IL-2 together with an inhibitor of TGF-β1 in nanoscale liposomal polymeric particles [13], pH-dependent release of IL-12 in the tumor bed to polarize macrophages from M2- to M1-type [14], therapeutic delivery of nanoparticular RNA-lipoplexes (RNA-LPXs) encoding IL-12 in a MYC oncogene-driven murine liver cancer [15], and protein nanogel-enhanced delivery of T cell activating IL-15 in a murine melanoma model and in a humanized glioblastoma mouse model treated with human CAR T cells [16]. More recently, Li and colleagues reported the successful elimination of established subcutaneous melanoma (B16F10 and YUMMER1.7 cell lines) or colon carcinoma (CT26 cell line) by virtue of intratumoral injection of ionizable lipid nanoparticles that were loaded with a self-replicating IL-12 RNA (IL-12 replicon) [17]. However, and despite high initial expectations, the lack of clinical translation of most nanoparticular cytokine delivery formats and the advent of immune checkpoint-blocking therapies have so far limited the further exploration and broad clinical use of cytokine-based nanoparticles.

### 2.2. Immune Checkpoint Inhibitors

The central role of ICIs in cancer immunotherapy was initially demonstrated in melanoma and non-small cell lung cancer patients by the remarkable clinical efficacy of antibodies that target cytotoxic T lymphocyte–associated antigen 4 (CTLA-4) and the programmed cell death protein 1 pathway (PD-1/PD-L1) [18,19]. While ICI therapy is proving to be effective and durable in a subset of patients with a variety of tumor types [20,21], only a fraction of patients show objective clinical responses and some responders eventually become refractory to ICI therapy [7]. The increasing clinical use of ICIs also revealed that treatment with CTLA-4 and PD-1/PD-L1 blocking antibodies may lead to severe immune-related adverse events, including gastrointestinal, pulmonary, hepatic, dermatological and endocrine toxicities, and autoimmunity [22]. A potential solution for improving efficacy and for reducing toxicity is to direct ICI activity to the tumor microenvironment (TME). In cases in which tumors are externally accessible, microneedle delivery can be very effective. One excellent example illustrating the therapeutic potential of microneedle nanotechnology is a study by Wang and colleagues who used this approach for tumor-associated anti-PD-1 antibody release in a subcutaneous B16F10 mouse melanoma model [23]. In this study, microneedle-mediated intra-tumoral injection prompted TME retention of the anti-PD1 antibody, resulting in a superior therapeutic immune response when compared with systemic injection of the anti-PD1 antibody. Microneedle lymphatic delivery can be used to direct ICI activity to secondary lymphatic organs, as demonstrated with anti-CTLA-4 blocking antibodies in a mouse model of breast cancer. This site-specific delivery increased tumor infiltrating lymphocytes, resulted in superior tumor growth inhibition, arrested metastatic spread, and induced complete anti-tumor responses when compared with systemic anti-CTLA-4 antibody administration. Moreover, the authors could demonstrate that this nanotopographical lymphatic infusion not only worked in mice, but also reached the axilla and the inguinal lymph nodes of healthy human volunteers [24], making this approach a promising technology for future clinical application.

In cases in which tumors are not externally accessible, ICI activity was locally concentrated by producing triblock copolymer-nanoparticles that comprise a central polyethylene glycol (PEG) block flanked by two polypeptide blocks, which contain reactive oxygen species (ROS)-responsive L-methionine (Me) and dextro-1-methyl-tryptophan (D-1MT) and tumor-homing high-affinity peptides derived from placental growth factor–2. The latter bind to fibronectin and collagen type I of the tumor extracellular matrix (ECM) [25]. These nanocarriers specifically released their cargo into the TME at low pH, as is prevalent in hypoxic tumors, and there induced high levels of tumor-toxic ROS via inhibition of indoleamine-2,3-dioxygenase (IDO-1) by D-1MT [26]. Local tumor responsiveness to ICIs can be further improved by including additional immune-enhancing cargos. For example, inclusion of a Toll-like receptor 9 (TLR9) immune-stimulating oligonucleotide into cysteine-linked tandem peptide nanocomplexes composed of an *N*-terminal myristoyl chain coupled to transportan, a cell penetrating peptide, and C-terminal peptides with a TME/TAM homing sequence (LyP1: cyclic *Cys-Gly-Asn-Lys-Arg-Thr-Arg-Gly Cys*, CRV: Cys-Arg-Val, RGD: Arg-Gly-Asp) dramatically improved treatment responsiveness to anti-CTLA-4, produced similar therapeutic benefits as 10- to 200-fold higher systemic delivery, and generated anti-tumor effects at distant tumor sites [27]. Similarly, poly-(β-amino ester) nanoparticle delivery of a stimulator of interferon genes (STING) signaling agonist (ADU S-100), when administered together with a PD-1 blocking antibody, resulted in a >10-fold improvement of anti-tumor effects in a B16 melanoma model [28].

Taken together, these pioneering experiments highlight that nanoparticle formulations have the potential to enhance tumor responsiveness to ICI therapy and to reduce unwanted systemic toxicity. Given their potential to fine-tune anti-cancer responses, site-specific nanoparticle delivery and co-packaging with additional immune-boosting agents is likely to advance future ICI-mediated anti-tumor treatments.

## 3. Personalized Anti-Cancer Therapies and Nanotechnology

In contrast to generalized immune-boosting therapies, personalized anti-cancer treatment redirects immune effector cells to patient-specific tumor vulnerabilities. To ensure that the patient is amenable for treatment, upfront knowledge of patient tumor characteristics and information about individual immunological predispositions are required. This means that, for most personalized therapies, critical variables such as individual tumor mutations, expression and presentation of cancer-specific neoantigens, the general immune status, and the nature of HLA alleles have to be tested before therapy can be started.

### 3.1. Adoptive Cell Transfer (ACT) of Lymphocytes

Because T cells are decisive effectors of cancer immunotherapy, treatment strategies that either use patient-derived tumor-reactive T cells, or alternatively, modify the reactive capacity of T cells towards tumor-specific antigens, have been developed. Initial experiments using re-infusion of autologous in vitro expanded tumor-infiltrating T lymphocytes (TILs) demonstrated durable complete responses in some cancer patients [29,30]. Moreover, the therapeutic action of such ACTs could be further improved with nanotechnology. A rational approach is to use tumor-localized implants loaded with tumor-specific lymphocytes that contain additional T cell-activating biologicals (such as activating anti-CD3, anti-CD28, and anti-CD137 antibodies) and T cell stimulatory cytokines (like IL-2 or IL-15). Preclinical experiments with TILs-containing nanoparticular implants that were also loaded with T cell-activating antibodies and cytokines confirmed that such nanoparticular formats have excellent tumor killing potential and are superior to locally applied TILs [31]. Such TIL-loaded nanoparticular implants can also be localized to sites of inoperable or only partially resectable tumor sites. However, and given the considerable complexity, costs, and technical effort required, ACT of tumor-reactive TILs has so far not become a standard-of-care in the treatment of cancer.

### 3.2. Patient-Derived Genetically Engineered T Cells (GETs)

An excellent alternative to re-infused TILs are GETs. The objective of GETs is to redirect the potent cytotoxic activity of T cells towards tumor-specific antigens. Of note is also that other lymphoid effector cells like natural killer (NK), natural killer T (NKT), and γδ T cells can be modified to recognize tumor-associated antigens and are equally proficient to eliminate cancer cells [32,33]. A crucial advantage over re-infusion of TILs is that GETs are engineered from T cells directly isolated from peripheral blood and can be produced in large numbers. Moreover, effective transduction of human T lymphocytes with viral expression vectors has become a safe procedure [34]. As of today, GETs come in two classes: The first class of GETs consists of genetically modified T cells that express a conventional T cell receptor (TCR). These TCR-GETs recognize an intracellularly processed peptide on the target cell in the context of a specific HLA haplotype. Once genetically engineered, such TCR-GETs rely on normal T cell signaling pathways, are subject to patient-specific HLA presentation differences, and have to deal with natural counter-acting mechanisms in the tumor able to reduce TCR signaling strength. The second class of GETs are T cells that express a recombinant chimeric antigen receptor (CAR). For antigen recognition, CARs normally use an antibody-derived extracellular single-chain variable fragment (scFv), which is linked to intracellular costimulatory domains (usually CD28 and/or 4-1BB), and to a cytoplasmic activation domain (CD3ζ or FcRγ) [3]. A crucial characteristic of CAR-expressing T cells is that they can recognize any target structure that is exposed on the cell surface. This means that CAR T cells are able to attack and destroy tumor cells independently of individual HLA haplotypes, thus making CAR therapy accessible to every patient. A second advantage of CAR T cells is their ability to eliminate weakly immunogenic cancer cells that have already lost HLA presentation, and thus have become “invisible” to normal T cells. Despite the documented objective responsiveness to TCR-GETs in some cancer patients [35] and the impressive success of CAR therapies in treating hematological malignancies [36,37,38], cancer immunotherapies with modified T cells still face several major challenges. These include a complex and expensive manufacturing process; life-threatening off-target effects, such as cytokine storm and graft-versus-host disease; and, in particular, poor in vivo CAR-T cell activity to solid tumors. In this regard, and as described in the following, nanotechnology can provide useful additional resources for overcoming these limitations.

### 3.3. Improving CAR T Cell Therapies by Nanotechnology

To develop a simple and inexpensive method for producing CAR T cells, Smith and collaborators synthesized poly-(β-amino ester) nanoparticles to transfect coding DNA in vivo specifically to T cells [39]. Most importantly, injection of these DNA-loaded nanoparticles into a mouse model of B-cell leukemia generated increasing numbers of active CAR T cells, induced efficient destruction of targeted cancer cells, and established long-lived CAR T memory cells. Although preclinical mouse data are often not fully transferable to human application, in vivo delivery of CAR-encoding nanoparticles to human T cells has the potential to greatly simplify CAR T cell generation and bring down the very high costs of ex vivo CAR T cell production and application for human use, amounting to 300,000–400,000 Euro for treatment of lymphoma [40].

Although very effective in destroying tumor cells, CAR T cell applications carry the above-mentioned risk of potentially life-threatening side effects [3,41]. Such adverse effects either manifest as severe on-target off-tumor destruction of healthy tissues that are wrongly recognized by the CAR, or are characterized by escalated general or neurotoxic immune responses known as cytokine release syndrome (CRS) and CAR-T-cell-related encephalopathy syndrome (CRES). One possible approach to make CAR therapy safer is to use nanotechnology for modulating CAR T cell specificity and activity. In first proof-of-principle experiments Albert and colleagues generated adaptor CAR-T cells (AdCARs, also called modCARs) that were completely dependent on the presence of nanoparticular adaptors [42]. The underlying principle of this technology is that AdCARs are unable to recognize the tumor target, but instead become activated by binding the recognition tag integrated into a camelid nanobody (single-domain antibody, consisting of only one single monomeric variable antibody domain) that recognizes and binds to the tumor antigen. By linking CAR T cell activity to the presence of a tagged tumor target-specific nanobody, the authors were able to efficiently control CAR T cell activity in tissue culture experiments and in mice. Most importantly and as binding of AdCARs is only dependent on the presence of the adaptor, their activity can be set off, fine-tuned, stopped, or switched to additional targets at any time during therapy. In a clinical setting, tight exogenous control over CAR T cell activity will be especially useful at the start of therapy, when unexpected off-target effects might take place and when patients are at higher risk of excessive general or neurotoxic immune responses.

In contrast to the very effective therapeutic action of CAR T cells in B cell leukemia, achieving powerful CAR T cell responses to destroy solid tumors remains a challenge. Obvious reasons for this are the poor persistence and tumor infiltration of adoptively transferred CAR T cells, the frequently encountered barriers of an immunosuppressive TME, and the suboptimal in vivo stimulation of CAR T cells by the tumor cells. To overcome these limitations and to be able to enhance CAR T cell activity, one possibility is to attach T cell boosting nanoparticles to their surfaces. Demonstrating the feasibility of such a strategy, the linking of liposomal vesicles loaded with an immune stimulatory A2a adenosine receptor antagonist (SCH-58261) to the surface of CAR T cells markedly improved the anti-cancer CAR T cell responses in mice with leukemia and ovarian cell carcinoma [43,44]. These experiments demonstrated that stable surface conjugation of nanoparticles does not necessarily compromise T cell effector function and reveals that attaching T cell-stimulating nanoparticles to CAR T cells fosters their activity in the TME and improves antitumor responses. A very good alternative for exogenously controlling and stimulating CAR T cell activity is nanoparticular RNA-lipoplexes (RNA-LPXs). Using this approach, Sahin and colleagues engineered splenic antigen presenting cell (APC)-targeted RNA-LPXs for expression of the cognate CAR T cell antigen in professional antigen presenting cells (APCs). Demonstrating the usefulness of this strategy, injection of these RNA-LPXs significantly improved CAR T cell efficacy and tumor destruction [45]. Of note, the same group successfully used tumor antigen-specific RNA-LPXs to trigger endogenous CD4 and CD8 T cell-mediated destruction of antigen-presenting tumor cells in mice and patients [46,47]. Thus, systemic delivery of tumor-specific RNA-LPX nanoparticles to APCs is a very efficient strategy for modulating and enhancing both endogenous and CAR T cell therapies and is a powerful new treatment option in personalized medicine.

### 3.4. Bi-Specific Engager Antibodies

An additional major area of research in precision immunotherapy is bi-specific antibodies, also known as bi-specific T cell engagers (BiTEs). In contrast to CARs that contain only one antibody-derived single-chain variable fragment (scFv) for target recognition, BiTEs have two different scFvs. The design of BiTEs allows binding of one scFv to a T cell epitope (often CD3ε) and the other to engage a tumor-associated surface antigen. By interacting with both targets, BiTEs physically link T cells and tumor cells, a bridging that results in T cell activation, cytokine production, and tumor destruction [48,49]. Accordingly, and owing to their potential for antigen-specific cell killing, a first BiTE therapy is available for the treatment of patients with relapsed and/or refractory B cell-precursor acute lymphoblastic leukemia [50]. However, and because most bi-specific antibodies suffer from short serum half-life times, to achieve optimal clinical efficacy, continuous infusion is required [51]. In addition, BiTE production for clinical use is costly and treatment can be associated with unwanted toxicities [49]. To test a novel strategy that circumvents laboratory production of BiTEs, Stadler and colleagues used nanoparticular RNA-LPXs to transiently express a tumor antigen-specific BiTE in mice [52]. Injection of RNA-LPXs facilitated sustained in vivo antibody expression and eliminated advanced ovary clear cell tumors models as effectively as the corresponding purified BiTE. It is also worthwhile to mention that multispecific antibody formats, recognizing more than two antigens can be generated [53], and that bi- or multispecific antibodies can be used to redirect additional immune cell types [54]. Although nanotechnology has only started to expand BiTE treatment options, it can be expected that nanoparticular formulations will soon be used to improve the delivery, bioavailability, safety, and efficacy of BiTE anti-cancer immunotherapies.

### 3.5. Neoantigen-Specific Vaccination

The availability of next generation mutanome sequencing (NGMS) in combination with bioinformatics tools that predict the likelihood of a mutated neoantigen to be presented on cancer cells allows the rapid and cost-effective production of customized anticancer vaccines [55,56]. A first possibility for activating neoantigen-reactive T cells is to use synthetic peptide vaccines [57]. Although peptide vaccination in concert with several immune adjuvants is safe, well tolerated, and able to trigger tumor-reactive T cells, the overall T cell response observed in initial peptide-based studies was limited. However, combining multiple neoantigen-specific peptides with a Toll-like receptor 3 (TLR3) and a melanoma differentiation-associated protein 5 (MDA-5) agonist was therapeutically successful [58]. Thus, of the six melanoma patients that were vaccinated, four showed no signs of tumor recurrence in a follow-up period of up to 32 months, providing a strong rationale for further refining this methodology. One logical approach would be to combine neoantigen-specific peptide vaccination with nanoparticular formulations [59]. For example, Luo and colleagues convincingly demonstrated the improved therapeutic potential of small (29 nm diameter) polymeric nanoparticles that were loaded with synthetic peptides [60]. This combination significantly improved peptide delivery to peripheral lymph nodes and antigen presentation, boosted antigen-specific T cell reactivity, and eradicated established subcutaneous tumors in immunocompetent C57BL/6 mice. The authors could also demonstrate a beneficial therapeutic synergy with ICI treatment and the long-term activation of memory T cells. These preclinical data strongly suggest that nanotechnology can further improve the antitumor efficacy of antigen-specific peptide vaccines, thus offering reasonable prospects for future clinical translation.

The second class of neoantigen-specific antitumor vaccines consist of customized RNA-LPXs loaded with synthetic RNAs expressing multiple patient-specific tumor neoepitopes [61,62]. Based on preclinical studies demonstrating the primary delivery of these RNA-LPX to APCs in the spleen and a robust reduction of established tumors [47,63], customized RNA-LPXs were tested in a first phase I clinical trial with advanced stage melanoma patients [46]. Importantly, and demonstrating the therapeutic potential of customized RNA-LPX vaccination, all 13 enrolled patients showed significantly reduced cumulative recurrent metastatic events and experienced sustained progression-free survival over a maximum period of up to 23 months. In addition, vaccination prevented relapse in eight patients and induced complete (five patients), partial (one patient), and mixed (one patient) responses. Because additional anti-PD1 antibody treatment of one initially fast progressing patient produced a complete response, customized RNA-LPX vaccination is likely to further benefit from PD-1/PDL-1 blockade. These pioneering studies clearly show the enormous potential of nanotechnology-driven neoantigen-specific antitumor vaccines. Finally, and expanding current clinical applications, the same group recently showed that the potent therapeutic benefit of RNA-LPX vaccination is not limited to tumor-specific neoantigens, but, in addition, can be applied to non-mutant tumor-associated antigens that are primarily expressed in melanoma cells [64].

## 4. Nano-Sensitizing the Tumor Microenvironment (TME)

The therapeutic benefit or failure to respond to cancer immunotherapies greatly depends on the TME [65,66]. TMEs not only consist of heterogeneous cancer cells, but also contain a variety of other components such as resident or tumor-infiltrating immune cells, for instance, macrophages (TAM), extracellular matrix (ECM)-producing (myo)fibroblasts, angiogenic endothelial cells, and vascular pericytes. For this reason, physical properties, immunological status, and metabolic conditions may vary significantly between individual TMEs and at metastatic sites. Although many individual factors in the TME can restrain therapy response, physical and functional exclusion of immune effector cells from the tumor bed and the immunosuppressive nature of the TME are major obstacles for cancer immunotherapy. A number of experimental and clinical studies, using various nanoparticle formats, have addressed these challenges. These studies demonstrated that combining cancer cell-targeted immunotherapy with TME-targeted and -responsive nanoparticles can overcome, at least in part, the limitations posed by the TME.

### 4.1. Overcoming the Physico-Chemical Barriers of the TME

**Figure 2:**
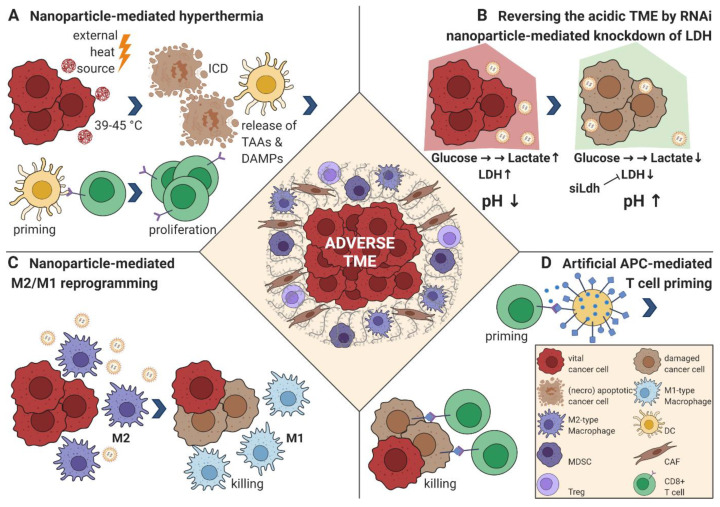
Strategies to reprogram the suppressive TME. (**A**) Targeted nanoparticles are administered and home to the TME. Upon external irradiation with an energy source (light, magnetic field, radiofrequency application), the surrounding tissue is heated to 39–45 °C, which induces cell stress that triggers the unfolded protein response (UPR) [79]. The UPR can lead to immunogenic cell death (ICD); the release of tumor-associated antigens (TAAs) and danger-associated molecular patterns (DAMPs); and activation of myeloid cells, especially dendritic cells (DCs), that prime T cells to initiate an antigen-specific adaptive anti-cancer immune response executed mainly by CD8^+^ cytotoxic T cells [70]. (**B**) RNA-binding nanoparticles are loaded with siRNA targeting lactate dehydrogenase A (LDH). The functional knockdown of LDH minimizes lactate production, reversing the acidic pH of the TME, with a subsequently enhanced immune response and a reduced tumor neoangiogenesis [75]. (**C**) Nanoparticles for TAM-specific delivery of TLR agonists [80,81] or small molecules inhibiting colony-stimulating factor 1 receptor (CSF-1R) [82] can shift the immune-suppressive M2 phenotype into an inflammatory M1 phenotype promoting CD8^+^ T cell mediated killing of cancer cells. (**D**) Artificial antigen presenting cells (aAPCs) express at least one peptide-MHC complex (for example, loaded with a highly expressed intracellular TAA) and a costimulatory signal, for example, anti-CD28 for effective T cell priming. They can also be further modified and loaded with cytokines or ICIs. In comparison with their cellular counterparts, they have the benefit of maintaining an “always on” state that cannot be inactivated [83], allowing for an effective anti-cancer CD8^+^ T cell priming in the otherwise immunosuppressive TME. Abbreviations: dendritic cell (DC), myeloid-derived suppressor cell (MDSC), cancer-associated fibroblast (CAF), regulatory T cell (Treg), siRNA directed to LDH (siLdh).

Compared with normal tissue, tumors develop an altered ECM, a tumor-specific neo-vasculature, and increased tissue pressure, all known to hamper homing, proliferation, and activation of immune effector cells [67,68]. An additional adverse effect is the acidic and hypoxic state of the TME and the associated metabolic changes. One approach to overcome the structural, functional, and physical roadblocks of the TME is immunogenic TME cell death (ICD), a process that not only kills cells, but also potentiates antigen-specific immune responses [69]. To direct ICD activity to the TME, either nano-carriers containing ICD-inducing chemotherapeutic drugs or tumor-homing nanoparticles, which locally generate hyperthermia upon external irradiation with an energy source, have emerged as promising combinatorial cancer immunotherapy options (Figure 2A) [70,71]. An alternative powerful approach for surmounting adverse structural and functional TME hurdles is to load nanoparticles with small interfering RNA (siRNA). The combination of immunotherapy with siRNA nanoparticles can efficiently silence otherwise “undruggable” genes in difficult to reach target sites, and thus specifically prime the TME for immunotherapy. The feasibility of such an approach was demonstrated by targeting the expression of the major ECM protein collagen type I with lipid-like and nanogel RNAi particles [72,73,74]. In these experiments, liver-specific delivery efficiently suppressed collagen synthesis by up to 95% and ameliorated hepatic fibrosis in mice. Similarly, and illustrating the manifold opportunities nanotechnology offers to sensitize the TME, Wang and colleagues showed that the adverse acidic milieu of the TME was reversed by RNAi nanoparticle-mediated knockdown of lactate dehydrogenase A, a process that restored antitumor T cell function and potentiated ICI therapy response (Figure 2B) [75]. Moreover, nanoparticles efficiently mitigating hypoxia, promoting TME perfusion, and disrupting the tumor vasculature have been generated [76]. Finally, Hou and colleagues could also address cancer-associated fibroblasts (CAFs) using a nanoemulsion of the potential antifibrotic compound fraxinellone in a mouse model of desmoplastic melanoma [77]. Targeting of CAFs might also have additional beneficial therapeutic effects because these cells not only secrete tumor sustaining ECM proteins like collagens, fibronectin, vitronectin, or laminins, or glycosaminoglycans and proteoglycans, for example, hyaluronan, but also synthesize tumor growth- and resistance-promoting growth factors as well as cytokines and chemokines suppressing the tumor immune response [78]. The above-discussed examples illustrate that nanoparticular formulations provide an excellent opportunity to reverse structural and functional barriers in the TME, and thus increase the susceptibility to cancer immunotherapeutic approaches against solid tumors.

### 4.2. Modulating Immune Suppressive Cells

Several tumor-associated cell types, most notably, regulatory T cells (Tregs), tumor-associated macrophages (TAMs), and myeloid-derived suppressor cells (MDSCs), act as negative regulators for cancer immunotherapy [84]. In the TME, these cells block cancer cell destruction by directly engaging immune effector cells or indirectly by secreting factors that establish immune tolerance. Several strategies have been adapted for nanoparticle-mediated targeting of these cells.

#### 4.2.1. Regulatory T Cells (Tregs)

Tregs are an important immune suppressive population in the TME and have the potential to inhibit cancer immunotherapy by numerous mechanisms [85,86]. These include, but are not restricted to, destruction of immune effector cells by granzyme and perforin; contact-dependent suppression of antigen-presenting cells; competition for IL-2, which is needed for proper effector T cell function; metabolic conversion of pro-inflammatory adenosine triphosphate (ATP) to immunosuppressive adenosine, especially by the ecto-nucleotidase CD39 [87]; and secretion of anti-inflammatory cytokines (IL-10, TGF-β1, and IL-35). For this reason, limiting Treg immune suppression is an obvious choice to enhance cancer immunotherapy. Because generalized blocking of Treg function increases the risk of severe autoimmune responses, selective depletion of Treg in the TME using nanotechnology can be particularly beneficial, as demonstrated by the selective depletion of Tregs using CD25-targeted near infrared photo-immunotherapy [88,89]. So far, TME-specific Treg-targeting of nanoparticles in combination with other immune therapies has not been tested, except for Ou et al., who demonstrated a synergistic effect when combining the selective delivery of Treg-specific tLyp1 peptide conjugated nanoparticles in the TME with ICIs [90]. The tLyp1 peptide is defined as a cell-penetrating peptide with the C-end rule motif R/KXXR/K that has a high affinity and specificity for the Neuropilin-1 (Nrp1) receptor [91,92,93]. The Nrp1 receptor can be found on many cancer types [94], but also on a majority of Tregs, where the expression even correlates with their survival and suppressive function [95]. When Imatinib within tLyp1 peptide-conjugated nanoparticles was combined with anti-CTLA-4 antibody, the authors found an enhanced downregulation of suppressive Tregs and activation of CD8^+^ effector T cells in the B16/B6 melanoma model [90].

#### 4.2.2. Tumor-Associated Macrophages (TAMs)

TAMs represent the prominent innate immune cell type that is paramount for tumor progression and cancer immunotherapy, apart from MDSC and tolerogenic M2-type DCs [96,97]. Depending on the exogenous cues existing in the TME, TAMs either exert pro-inflammatory tumoricidal activities (M1-type TAMs) or adapt an anti-inflammatory and wound-healing phenotype, which supports tumor progression (M2-type TAMs) [98]. Reflecting their importance for tumor evolution, TAMs account for more than 50% of the tumor mass in some solid tumors and can regulate ECM synthesis, vascular remodeling, cell motility, invasion, and extravasation. In addition, TAMs suppress effector T and NK cell function, recruit Tregs to the TME, and interact with neighboring fibroblasts to promote tumor progression [99]. Given their abundance, their regulatory function, and their specific phagocytic capacity, TAMs represent excellent cellular targets for nanoparticular delivery. Recent evidence suggests that nanoparticles can be directed to tumor-resident TAMs and, depending on the nanoparticular formulation, be utilized to deplete or repolarize M2 TAMs (Figure 2C) [82,100]. First preclinical data in mouse tumor models combining nanoparticular M2 targeting with immunotherapy provide grounds for optimism. For example, Huang and colleagues combined CpG oligonucleotides (ODN), anti-IL-10 ODN and anti-IL-10RA ODN, with galactosylated cationic dextran, which binds the ODN promoting the formation of stable nano-complexes and directs the nanoparticles to galactose-type lectin (MgI), which is highly expressed on TAMs. These stable nano-complexes were further packaged into a pH-sensitive PEG-histidine-modified alginate to specifically release the nano-complex in the acidic TME. Application of these nanoparticles repolarized M2 TAMs to an M1 phenotype, reduced tumor angiogenesis, and was very effective at suppressing tumor growth [80]. Several additional studies exploited nanoparticular TAM delivery for reprogramming the TME towards a more immunogenic state such as TAM-depleting bisphosphonates, TAM-repolarizing TLR agonists, co-delivery of immune adjuvants with target antigens, siRNA-mediated inhibition of immunosuppressive pathways, and the targeting of ICD-inducing chemotherapeutic drugs [96,101].

#### 4.2.3. Myeloid-Derived Suppressor Cells (MDSCs)

The presence of MDSCs that are recruited from the blood stream into the TME constitutes a major obstacle for immunotherapy [102]. Normally required to restore tissue homeostasis following infection and traumatic stress, MDSC progenitors fail to differentiate into immunogenic myeloid DCs and inflammatory macrophages in the TME [103]. Once recruited, MDSCs promote aberrant tumor vascularization, disrupt antigen presentation by dendritic cells (DCs), suppress T and NK cell function, induce Treg activity, and polarize TAMs to an M2 phenotype [104]. Direct evidence that “exclusion”, “killing”, or “re-education” of MDSCs with nanoparticles can ameliorate the suppressive immune contexture of TMEs has been provided by several studies. To prevent MDSC progenitors from homing into the TME, Leuschner and colleagues developed a 70–80 nm lipid-like particle containing C-C chemokine receptor type 2 (CCR2)-specific siRNA. Because CCR2 is required for guiding MDSC-precursors (and monocytes-macrophages) to their target tissues, systemic application of CCR2 siRNA nanoparticles reduced the number of MDSCs in the TME and decreased the tumor volume as monotherapy [101]. Given the synergistic benefits of PF-04136309, a small molecule CCR2 inhibitor, when administered together with chemotherapy in locally advanced pancreatic cancer patients [105], there is a good chance that nanoparticles directing chemokine receptor blocking agents to the bone marrow and the spleen might improve responses to cancer immunotherapy via blocking the recruitment of tumor infiltrating MDSC. The second alternative to condition the TME for immunotherapy is to utilize nanoparticles capable of MDSC depletion or repolarization. First preclinical experiments demonstrated that MDSC depleting nanoparticles, specifically nanoscale polymeric micelles releasing 6-thioguanine; high-density lipoprotein-like nanoparticles specifically targeting scavenger receptor type B on MDCSs; or lipid-coated biodegradable hollow mesoporous silica nanoparticles co-encapsulating all-trans retinoic acid, doxorubicin, and IL-2, efficiently reduced MDSC numbers or activity in the TME and increased immune effector activity [106,107,108].

### 4.3. Nanoscale Artificial Antigen Presenting Cells for Cancer Immunotherapy (aAPCs)

One emerging new principle to enhance the efficacy of cancer immunotherapies is aAPCs [109]. These nano- or microsized particles are composed of, for example, polystyrene, biodegradable poly-glycolic acid (PGA), or poly-lactic-co-glycolic acid (PLGA), and are designed to provide physiologically relevant signals that are needed for target recognition and destruction by CD8^+^ T cells (Figure 2D). These aAPCs consist of at least a peptide-major histocompatibility complex (MHC) conjugate presenting an abundant tumor associated antigen (TAA), and a costimulatory signal delivered, for example, via an activating anti-CD28 antibody resulting in efficient T cell activation, but can additionally harbor cytokines to be released in the TME [83]. Major advantages associated with aAPCs are their capability to infiltrate even difficult-to-reach TMEs and metastatic areas, the potential to prime effector T cells for specific tumor antigens, and a complete insensitivity to signals that might otherwise restrict the capability of conventional APCs. First promising in vivo experiments in mice demonstrated that combining aAPC technology with anti-PD-1 ICI immunotherapy synergistically stimulated antigen-specific proliferation of cytotoxic T cells, delayed murine melanoma growth, and extended survival, while either treatment alone had no effect [83]. Equally, injection of aAPC particles loaded with both IL-2 and anti-CTLA-4 outperformed conventional single-release aAPCs, efficiently promoted sustained ICI and IL-2 release as well as antigen-specific T cell activation and expansion, and inhibited tumor growth in a murine melanoma model [110]. To make aAPCs even more versatile, Zhang and colleagues developed a multipotent artificial antigen-presenting cell (MaAPC) [111]. This MaAPC displayed the “self-marker” CD47-Fc to prevent macrophage phagocytosis and premature clearance; stimulated effector T cells and established T cell memory with three costimulatory molecules (anti-CD28, anti–4-1BB, and anti-CD2); directed T cell killing against two tumor antigens; and released IL-2, IL-15, and CCL21 together with anti-CTLA-4 and anti-PD-1 ICIs. Accordingly, this “supercharged aAPC” efficiently inhibited subcutaneous melanoma growth in a mouse model, massively expanded target antigen specific cytotoxic T cells over 200-fold in tumor tissues, and established a cytokine profile favorable for tumor cell destruction [111]. Most importantly, no immune toxicities were detected with these three different aAPC combination therapies. Although we do not know how cancer patients will react to aAPC-therapies, the strong synergistic anti-cancer effect produced in mice raises great expectations for the future.

## 5. Conclusions and Outlook

Given the advances in nanotechnology and the clinical success of cancer immunotherapy, the convergence of these two disciplines will surely generate substantial momentum for improving cancer treatment. Today, it is not evident if nanomaterials are the ultimate rationale for making immunotherapies work, but they have great potential to provide good solutions for overcoming the current limitations of cancer immunotherapy. In particular, nanotechnology can improve on-target delivery to specific tissues, cells, and subcellular compartments; ensure sustained stability and release; reduce dose-limiting toxicities; and mitigate adverse effects imposed by the TME. However, there exist important caveats and potential pitfalls that have to be considered and overcome. From a therapeutic standpoint, the key question is as follows: How many vulnerabilities must we target and what are the best combinations to improve the clinical outcome in patients undergoing immune-based therapy? It will also be important to ask how faithful animal experiments translate to patients. In addition, non-toxic nanomaterials suitable for human application must be designed and confirmed in vivo. To ensure actionable therapeutic solutions that successfully combine cancer immunology and nanotechnology, it will also be necessary to develop reliable and predictive biomarkers and to understand mechanisms of resistance (recently reviewed in [112,113]). As attempts to improve cancer immunotherapies, especially when combined with nanoparticular approaches, are exceedingly complex and might initially fail, special attention should be given to the optimization of in vitro and in vivo preclinical models to streamline efficacy testing, and to appropriate biomarkers that permit to non-invasively assess early anti-tumor immune responses in phase 1–2 clinical studies.

## Abbreviations

ACT	adoptive cell transfer
APC	antigen presenting cell
aAPC	artificial antigen presenting cell
AdCAR	adaptor CAR-T cell
BiTE	bi-specific T cell engager
CAR	chimeric antigen receptor
CSF-1R	colony-stimulating factor 1 receptor
CTLA-4	cytotoxic T lymphocyte-associated antigen 4
DAMP	danger-associated molecular pattern
ECM	extracellular matrix
GETs	genetically engineered T cells
HLA	human leukocyte antigen
ICD	immunogenic cell death
ICI	immune checkpoint inhibitor
IL-2	interleukin-2
LDH	lactate dehydrogenase A
MDA-5	melanoma differentiation-associated protein 5
MDSC	myeloid-derived suppressor cell
NGMS	next generation mutanome sequencing
PD-1/PD-L1	programmed cell death protein 1 pathway
PEG	polyethylene glycol
RNA-LPX	RNA-lipoplex
ROS	reactive oxygen species
scFv	single-chain variable fragment
siRNA	small interfering RNA
TAA	tumor-associated antigen
TAM	tumor-associated macrophage
TCR	T cell receptor
TGF-β1	transforming growth factor-β1
TIL	tumor-infiltrating T lymphocyte
TLR	toll-like receptor
TME	tumor microenvironment, Treg, regulatory T cell
TAA	tumor-associated antigen
UPR	unfolded protein response
